# Genetic variation and possible origins of weedy rice found in California

**DOI:** 10.1002/ece3.5167

**Published:** 2019-04-22

**Authors:** Teresa B. De Leon, Elizabeth Karn, Kassim Al‐Khatib, Luis Espino, Timothy Blank, Cynthia B. Andaya, Virgilio C. Andaya, Whitney Brim‐DeForest

**Affiliations:** ^1^ Department of Plant Sciences University of California, Davis Davis California; ^2^ Rice Experiment Station California Cooperative Rice Research Foundation, Inc. Biggs California; ^3^ Cooperative Extension Sutter‐Yuba Counties University of California Division of Agricultural and Natural Resources (UC ANR) Yuba City California; ^4^ Cooperative Extension Colusa County University of California Division of Agricultural and Natural Resources (UC ANR) Colusa California; ^5^ California Crop Improvement Association University of California, Davis Davis California

**Keywords:** California, de‐domestication, hybridization, microsatellite markers, weedy rice

## Abstract

Control of weeds in cultivated crops is a pivotal component in successful crop production allowing higher yield and higher quality. In rice‐growing regions worldwide, weedy rice (*Oryza sativa* f.* spontanea* Rosh.) is a weed related to cultivated rice which infests rice fields. With populations across the globe evolving a suite of phenotypic traits characteristic of weeds and of cultivated rice, varying hypotheses exist on the origin of weedy rice. Here, we investigated the genetic diversity and possible origin of weedy rice in California using 98 simple sequence repeat (SSR) markers and an *Rc* gene‐specific marker. By employing phylogenetic clustering analysis, we show that four to five genetically distinct biotypes of weedy rice exist in California. Analysis of population structure and genetic distance among individuals reveals diverse evolutionary origins of California weedy rice biotypes, with ancestry derived from *indica*, *aus*, and *japonica* cultivated rice as well as possible contributions from weedy rice from the southern United States and wild rice. Because this diverse parentage primarily consists of weedy, wild, and cultivated rice not found in California, most existing weedy rice biotypes likely originated outside California.

## 1 INTRODUCTION

Plant domestication is the process of conversion of wild plants into domesticated crop plants through artificial selection. Domestication leads to plant varieties that are distinctly different from their wild ancestor, typically selecting for a suite of traits including the ability to grow in densely planted environments, easily controlled mating, reduced seed dormancy and seed shattering, good nutritive or fiber quality, and adaptation to local growing conditions (Gepts, 2004; Meyer & Purugganan, 2013). However, in the process, some cultivated plants may escape cultivation and evolve in unintended ways. Plant de‐domestication is an evolutionary process separate from domestication, in which plants develop genetically and phenotypically distinct “feral” populations through hybridization and/or adaptation (Ellstrand et al., 2010; Gressel, 2005). During de‐domestication, weedy populations typically re‐acquire traits that were lost in the process of domestication such as seed shattering and seed dormancy while retaining some crop‐specific traits such as crop growth form and ability to thrive in a densely planted managed field environment (Ellstrand et al., 2010; Gressel, 2005). These weedy populations pose a problem for crop cultivation because they are often similar enough to the crop to escape weed management practices, but can reduce the yield and value of the crop. Compared to the evolutionary process of domestication, de‐domestication is far less well understood, especially in terms of how these de‐domesticated plants came to be and how their populations and genomes are evolving (Ellstrand et al., 2010; Qiu et al., 2017; Wedger & Olsen, 2018).

Asian domesticated rice (*Oryza sativa* L.) originated in South and Southeast Asia from the wild rice species *Oryza rufipogon* Griff. Several distinct types of cultivated rice, including *japonica*, *indica*, and *aus* varieties, have evolved through multiple domestication events, adaptation to environment and rice‐growing practices, and selection for agronomic and culinary traits (Londo, Chiang, Hung, Chiang, & Schaal, 2006). Cultivated rice has been a model genetic system for agricultural plants because of its small genome, ease of genetic manipulation, and importance as a food source globally (Wedger & Olsen, 2018). This has led to a wealth of developed genetic resources, which can be used for the study of plant evolution. In the United States, two major rice‐growing regions, the Mississippi River flood plain in the southern United States and the Sacramento Valley region of California, produce tropical *japonica* and temperate *japonica* rice, respectively.

**Figure 1:**
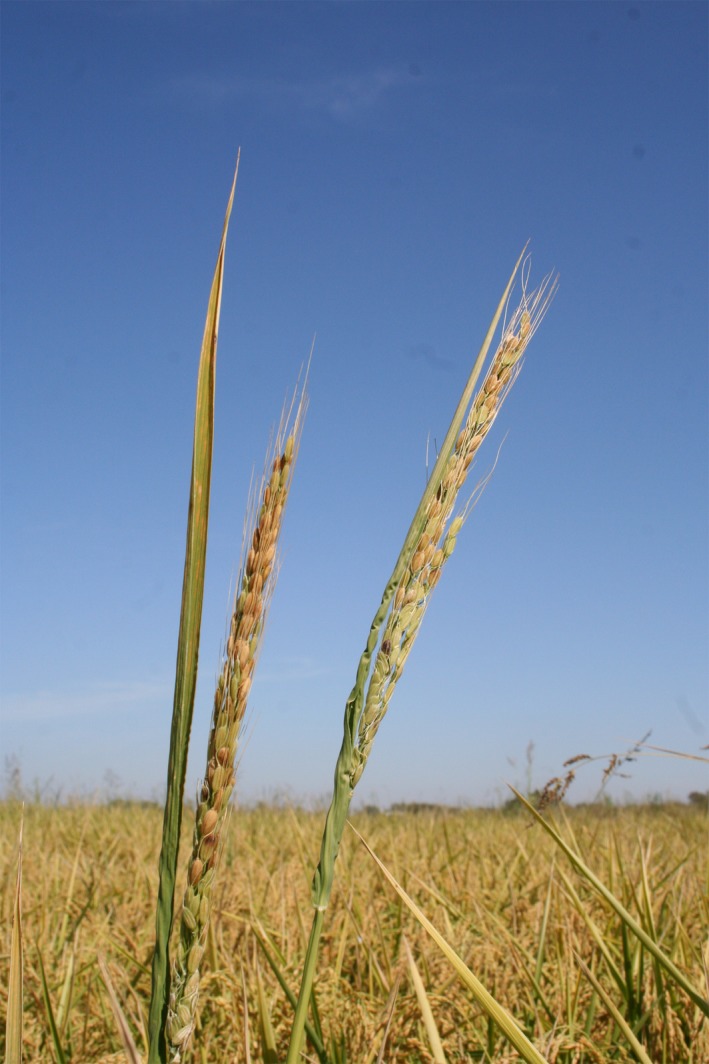
Weedy rice panicles in a field in Colusa County, California (photo credit: Luis Espino)

Weedy rice (*O. sativa* f.* spontanea* Rosh.), also known as red rice, is a major problematic weed of rice agriculture in many regions (Wedger & Olsen, 2018; Figure 1). It is considered to be the same species as cultivated rice, *O. sativa* (Langevin, Clay, & Grace, 1990). While populations of weedy rice vary, it can generally be distinguished from cultivated rice by the red seed pericarp it is named for, high seed shattering, and increased seed dormancy (Gealy, 2005). This weed competes with cultivated rice in the field, leading to yield losses of up to 49% in the southern United States (Shivrain et al., 2009). Weedy rice is phenotypically similar to cultivated rice during the vegetative stage, making it difficult to identify until late in the growing season. The phenotypic and biological similarities of weedy rice with cultivated rice make it difficult to control in‐season with either hand‐weeding or chemical weed control methods. Because weedy rice is conspecific with cultivated rice, the abundant genetic resources developed for cultivated rice can also be applied to weedy rice. With diverse populations found in rice‐growing regions around the world, weedy rice can be used as a model system to study weedy plant evolution as well as understanding the process of de‐domestication.

As early evolutionary biologists considered the origins of weeds that are related to crops, various hypotheses were proposed for evolutionary pathways to weediness (Baker, 1974; De Wet & Harlan, 1975; Ellstrand et al., 2010). Weedy rice populations across the globe offer support for many of these hypotheses. The endoferal hypothesis states that weedy crop relatives are derived directly from the crop as a result of de‐domestication (Gressel, 2005). Local weedy populations could have descended either from locally grown cultivars or from distantly located cultivars transported through movement of contaminated seed. Endoferal weedy rice populations that likely originated from local rice varieties have been identified in China (Cao et al., 2006; Xia, Wang, Xia, Zhao, & Lu, 2011). Some of these weed populations may have arisen through hybridization of *indica* and *japonica* cultivars, followed by environmental adaptation (Qiu et al., 2014). Weedy rice populations from the southern United States have been found to be descended from Asian *indica* and *aus* rice cultivars not grown in the United States (Londo & Schaal, 2007; Reagon et al., 2010). New weedy rice biotypes may also arise by hybridization of cultivated rice with existing weedy rice biotypes. Some populations of weedy rice in the southern United States originally derived from Asian cultivated sources have hybridized with each other and with local cultivars resulting in distinct weedy rice populations (Reagon et al., 2010; Shivrain et al., 2010). DNA sequencing of southern United States weedy rice and Asian weedy rice revealed that some populations contain a functional allele of the *Rc* gene responsible for both red pericarp and increased seed dormancy, indicating possible relationship to wild rice or Asian rice landraces never selected for white pericarp (Gross et al., 2010; Li, Li, Jia, Caicedo, & Olsen, 2017; Subudhi et al., 2012).

In contrast to the endoferal hypothesis, the exoferal hypothesis states that weedy populations are the result of hybridization of the crop with its wild relative (Ellstrand et al., 2010; Gressel, 2005), in the case of rice most likely with *O. rufipogon* or *O. nivara*. Domesticated rice and its wild ancestor *O. rufipogon* have some reproductive barriers, but gene flow is possible between domesticated, weedy, and wild rice (Bah, Merwe, & Labuschagne, 2017; Chu & Oka, 1970; Gealy, Mitten, & Rutger, 2003; Langevin et al., 1990). Some studies have proposed that some southern United States weedy rice populations evolved from crop–wild hybridization in China (Kanapeckas et al., 2016; Londo & Schaal, 2007), although there is limited empirical evidence for this. One final hypothesis is that weedy rice populations may not be derived from cultivated rice at all, but rather derived directly from wild rice species such as *O. rufipogon* or *O. nivara*, and that these populations have adapted to the environment of the cultivated rice agroecosystem, evolving phenotypic similarity with cultivated rice while retaining the seed shattering and seed dormancy traits of the wild species (Gressel, 2005). Some south Asian populations of weedy rice are likely descended from a wild rice ancestor (Huang et al., 2017). All of these hypotheses for the origins of de‐domesticated populations are nonmutually exclusive, and it is possible that a weedy population may have genetic contributions from several wild, weedy, or domesticated ancestors.

It is clear from these previous studies that weedy rice around the world has evolved through several independent origins from diverse sources. The suite of phenotypic traits characteristic of weedy rice has evolved multiple times through convergent evolution of diverse genetic mechanisms (Li et al., 2017; Qi et al., 2015; Qiu et al., 2017; Thurber, Jia, Jia, & Caicedo, 2013). These studies highlight the need to investigate the evolutionary origins of weedy rice in individual regions to gain a greater understanding of weedy rice evolution as a whole (Wedger & Olsen, 2018).

In California, weedy rice was reported in the early 20th century shortly after the beginning of commercial rice production and was hypothesized to have originated from contaminated seed from the southern United States (Bellue, 1932). In the 1950s, weedy rice was thought to be eradicated. The use of a continuously flooded system and the widespread adoption of a certified seed program involving third‐party field inspections and rice variety certification were credited as the reasons for the disappearance of weedy rice. In 2003, however, weedy rice of a single biotype was reported in a dry‐seeded rice field (Kanapeckas et al., 2016). Since then, weedy rice has been identified in at least 60 fields and on over 4,050 ha in 2016 (Whitney Brim‐DeForest, personal communication, February 5, 2018). While weedy rice in the southern United States has been well‐characterized (Li et al., 2017; Londo & Schaal, 2007; Reagon et al., 2010), weedy rice in California is a recent and growing problem with previous studies limited to one or two biotypes (Kanapeckas et al., 2017,2016; Londo & Schaal, 2007). It is unclear whether California weedy rice is derived from the weedy rice present in the southern United States, from cultivated rice inside or outside of California, from Asian wild rice, or from hybridization of any of these groups.

In this study, we seek to investigate the genetic diversity and relationships of California weedy rice, in order to gain insights into its evolutionary origins. We used microsatellite (SSR) markers and a *Rc* gene‐specific marker to genotype 48 California accessions of weedy rice, as well as weedy rice from the southern United States, wild rice, and cultivated rice at 99 loci. We used phylogenetic, population structuring, and genetic distance‐based approaches to examine possible relationships and evolutionary hypotheses for the origin of California weedy rice. We hypothesized that genetic diversity of weedy rice biotypes and their relationships to other rice groups would indicate multiple independent evolutionary origins of California weedy rice.

## 2 METHODS

### 2.1 Plant material

Weedy, wild, and cultivated rice samples selected for genotyping analysis totaled 96 samples. Forty‐six weedy rice samples from California included 18 accessions collected in 2006 and 28 samples collected in 2016 (Table 1). Samples were obtained from commercial rice fields in five of the nine major rice‐producing counties (Glenn, Colusa, Butte, Yuba, and Sutter counties) in the northern Sacramento Valley region of California. The majority of the 2006 collections were strawhull awned type or bronzehull awnless type, while several phenotypic types were present in 2016 collections (Tables 1 and 2). Four of the 2006 accessions were also used to produce plant material for other studies (Kanapeckas et al., 2017, 2016). To enable comparison with other weedy and wild rice, we included 20 weedy rice accessions from the southern United States (Arkansas, Mississippi, Missouri, Louisiana, and Texas) and 8 wild rice accessions. We also included a total of 22 cultivated rice accessions: 6 temperate *japonica*, 4 tropical *japonica*, 5 *indica*, 5 *aus*, 1 aromatic group V, and 2 red‐pericarp specialty rice accessions. Samples of cultivated rice, southern weedy rice, and wild rice were obtained from USDA collections and from the Rice Experiment Station (Biggs, California) (Table 1).

**Table 1:** List of rice genotypes used in the genetic study, (genotype source, grain attributes, and presence or absence of 14‐basepair deletion in *Rc* gene)

Sample ID	Biotype	County/Source	Pericarp color	Hull color	Grain type	Presence of awn	*Rc* gene deletion
RR2	Weedy rice	Glenn, CA (2006)	Red	Straw	MG	Awned	Absent
RR15	Weedy rice	Glenn, CA (2006)	Red	Straw	MG	Awned	Absent
RR28	Weedy rice	Glenn, CA (2006)	Red	Straw	MG	Awned	Absent
RR73	Weedy rice	Glenn, CA (2006)	Red	Straw	MG	Awned	Absent
RR76	Weedy rice	Sutter, CA (2006)	Red	Bronze	MG	Awnless	Absent
RR78	Weedy rice	Sutter, CA (2006)	Red	Bronze	MG	Awnless	Absent
RR80	Weedy rice	Colusa, CA (2006)	Red	Straw	MG	Awned	Absent
RR81	Weedy rice	Colusa, CA (2006)	Red	Straw	MG	Awned	Absent
RR90	Weedy rice	Glenn, CA (2006)	Red	Bronze	SG	Awnless	Absent
RR93	Weedy rice	Glenn, CA (2006)	Red	Bronze	MG	Awnless	Absent
RR95	Weedy rice	Glenn, CA (2006)	Red	Bronze	MG	Awnless	Absent
RR96	Weedy rice	Glenn, CA (2006)	Red	Bronze	SG	Awnless	Absent
RR97	Weedy rice	Glenn, CA (2006)	Red	Bronze	MG	Awnless	Absent
RR98	Weedy rice	Glenn, CA (2006)	Red	Bronze	MG	Awnless	Absent
RR99	Weedy rice	Glenn, CA (2006)	Red	Bronze	MG	Awnless	Absent
RR100	Weedy rice	Glenn, CA (2006)	Red	Bronze	MG	Awnless	Absent
RR101	Weedy rice	Glenn, CA (2006)	Red	Bronze	MG	Awnless	Absent
RR103	Weedy rice	Glenn, CA (2006)	Red	Bronze	SG	Awnless	Absent
RR104	Weedy rice	Butte, CA (2016)	Red	Bronze	MG	Awnless	Absent
RR105	Weedy rice	Butte, CA (2016)	Red	Straw	SG	Awnless	Absent
RR106	Weedy rice	Butte, CA (2016)	Red	Straw	SG	Awnless	Absent
RR107	Weedy rice	Butte, CA (2016)	Red	Straw	SG	Awnless	Absent
RR108	Weedy rice	Colusa, CA (2016)	Red	Straw	MG	Awned	Absent
RR109	Weedy rice	Colusa, CA (2016)	Red	Straw	MG	Awned	Absent
RR110	Weedy rice	Colusa, CA (2016)	Red	Straw	MG	Awned	Absent
RR111	Weedy rice	Glenn, CA (2016)	Red	Black	SG	Awned	Absent
RR112	Weedy rice	Glenn, CA (2016)	Red	Straw	MG	Awned	Absent
RR113	Weedy rice	Glenn, CA (2016)	Red	Straw	SG	Awnless	Absent
RR114	Weedy rice	Sutter, CA (2016)	Red	Bronze	MG	Awnless	Absent
RR115	Weedy rice	Sutter, CA (2016)	Red	Straw	SG	Awnless	Absent
RR116	Weedy rice	Sutter, CA (2016)	Red	Straw	SG	Awnless	Absent
RR117	Weedy rice	Sutter, CA (2016)	Red	Straw	SG	Awnless	Absent
RR118	Weedy rice	Sutter, CA (2016)	Red	Bronze	MG	Awnless	Absent
RR119	Weedy rice	Sutter, CA (2016)	Red	Straw	MG	Awnless	Absent
RR120	Weedy rice	Sutter, CA (2016)	Red	Straw	SG	Awnless	Absent
RR121	Weedy rice	Sutter, CA (2016)	Red	Straw	MG	Awnless	Absent
RR122	Weedy rice	Sutter, CA (2016)	Red	Straw	SG	Awnless	Absent
RR123	Weedy rice	Yuba, CA (2016)	Red	Straw	MG	Awnless	Absent
RR124	Weedy rice	Yuba, CA (2016)	Red	Bronze	MG	Awnless	Absent
RR125	Specialty Rice	CA (2016)	Red	Bronze	MG	Awnless	Absent
RR126	Specialty Rice	CA (2016)	Red	Straw	MG	Awnless	Absent
RR127	Weedy rice	Butte, CA (2016)	Red	Straw	LG	Awnless	Absent
RR128	Weedy rice	Sutter, CA (2016)	Red	Straw	LG	Awned	Absent
RR129	Weedy rice	Butte, CA (2016)	Red	Straw	MG	Awnless	Absent
RR130	Weedy rice	Butte, CA (2016)	Red	Straw	LG	Awned	Absent
RR131	Weedy rice	Butte, CA (2016)	Red	Straw	LG	Awnless	Absent
RR132	Weedy rice	Butte, CA (2016)	Red	Straw	SG	Awnless	Absent
RR133	Weedy rice	Butte, CA (2016)	Red	Straw	MG	Awnless	Absent
ruf	*O. rufipogon*	CCRRF	Red	Black	SG	Awned	Absent
wr1	*O. alta*	USDA (GSOR 311686)	Red	Black	LG	Awned	Absent
wr4	*O. nivara*	USDA (GSOR 311698)	Red	Black	MG	Awned	Absent
wr5	*O. nivara*	USDA (GSOR 311699)	Red	Bronze	MG	Awned	Absent
wr6	*O. officinalis*	USDA (GSOR 311700)	Light Brown	Straw	SG	Awnless	Ambiguous
wr7	*O. officinalis*	USDA (PI 590412 Ph)	White	Straw	MG	Awnless	Ambiguous
wr8	*O. nivara*	USDA (PI 590410 USA)	Red	Black	SG	Awnless	Absent
wr10	O. spp (wild)	USDA (GSOR 311791)	White	Straw	SG	Awnless	Present
ar1	Weedy rice—Arkansas	USDA (AR‐1994‐16B)	Red	Straw	MG	Awnless	Absent
ar2	Weedy rice—Arkansas	USDA (AR‐1994‐18A)	Red	Black	SG	Awned	Absent
ar3	Weedy rice—Arkansas	USDA (AR‐1995‐StgS)	Red	Straw	SG	Awnless	Absent
ar4	Weedy rice—Arkansas	USDA (AR‐2001‐1091)	Red	Straw	SG	Awnless	Absent
ar5	Weedy rice—Arkansas	USDA (AR‐2001‐1096)	Red	Black	SG	Awned	Absent
ar6	Weedy rice—Arkansas	USDA (AR‐2001‐1196)	Red	Straw	SG	Awnless	Absent
ar7	Weedy rice—Arkansas	USDA (AR‐2001‐1118)	Red	Straw	SG	Awnless	Absent
ar8	Weedy rice—Arkansas	USDA (AR‐2001‐1134)	Red	Straw	SG	Awnless	Absent
ar9	Weedy rice—Arkansas	USDA (AR‐2001‐1135)	Red	Straw	MG	Awnless	Absent
ar10	Weedy rice—Arkansas	USDA (AR‐2001‐1141)	Red	Straw	MG	Awnless	Absent
ms1	Weedy rice—Mississippi	USDA (MS‐1995‐15)	Red	Straw	MG	Awnless	Absent
ms2	Weedy rice—Mississippi	USDA (MS‐1995‐MS4)	Red	Bronze	MG	Awned	Absent
ms3	Weedy rice—Mississippi	USDA (MS‐1996‐5)	Red	Straw	SG	Awnless	Absent
ms4	Weedy rice—Mississippi	USDA (MS‐1996‐9)	Red	Black	MG	Awned	Absent
ms5	Weedy rice—Mississippi	USDA (MS‐2001‐1179)	Red	Bronze	MG	Awnless	Absent
mo1	Weedy rice—Missouri	USDA (MO‐2001‐1004)	Red	Straw	MG	Awnless	Absent
mo2	Weedy rice—Missouri	USDA (MO‐2001‐1098)	Red	Straw	MG	Awnless	Absent
la1	Weedy rice—Louisiana	USDA (LA‐2001‐1160)	Red	Straw	MG	Awnless	Absent
la2	Weedy rice—Louisiana	USDA (LA‐2001‐1188)	Red	Straw	SG	Awnless	Absent
tx1	Weedy rice—Texas	USDA (TX‐1995‐TX4)	Red	Black	MG	Awned	Absent
cav9	Calrose	CCRRF	White	Yellow/gold	MG	Awned	Present
cav8	M‐205	CCRRF	White	Yellow/gold	MG	Awned	Present
cav7	M‐202 (PI 494105)	CCRRF	White	Yellow/gold	MG	Awned	Present
cav4	Calmochi‐101	CCRRF	White	Yellow/gold	SG	Awnless	Present
cav14	NFD 109	USDA	White	Yellow/gold	SG	Awned	Present
cav20	Cal4810A1‐3	USDA	Red	Straw	MG	Awned	Absent
Temj6	Kagama Dango Mochi	USDA	Red	Straw	SG	Awned	Absent
tj1	Lemont	USDA	Light Brown	Yellow/gold	LG	Awnless	Present
tj2	Bengal	USDA	Light Brown	Yellow/gold	MG	Awnless	Present
tj5	Bluebonnet 50	USDA	Light Brown	Yellow/gold	LG	Awned	Present
in1	IR64	USDA	White	Straw	LG	Awned	Present
in2	IR29	USDA	White	Straw	LG	Awnless	Present
in3	Milagrosa	USDA	Light Brown	Straw	MG	Awned	Ambiguous
in4	Basmati	USDA	Light Brown	Straw	SG	Awnless	Absent
in5	Pokkali	USDA	Red	Straw	SG	Awned	Absent
au1	BJ‐1	USDA	Red	Bronze	SG	Awned	Absent
au2	Phudugey	USDA	Light Brown	Straw	MG	Awnless	Absent
au3	Kasalath	USDA	Red	Straw	SG	Awned	Absent
au4	Jhona‐349	USDA	Brown	Straw	MG	Awnless	Absent
au5	Dhala_Shaitta	USDA	Brown	Straw	MG	Awnless	Ambiguous

Note

CCRRF: California Cooperative Rice Research Foundation, Inc.; LG: long grain; MG: medium grain; SG: short grain; USDA: United States Department of Agriculture.

**Table 2:** Descriptions of California weedy rice biotypes based on hull color, presence of awns, and grain type

Biotype	Hull color	Awns	Grain type
Type 1	Strawhull	Absent	Short grain
Type 2	Bronzehull	Absent	Medium grain
Type 3	Strawhull	Long	Medium grain
Type 4	Blackhull	Long	Short grain
Type 5	Strawhull	Partially awned or absent	Medium or long grain

### 2.2 Genetic analysis

Genomic DNA was extracted from a 4‐cm‐long piece of leaf tissue from each plant sample using a modified TE‐potassium acetate extraction protocol (Tai & Tanksley, 1990). Extracted genomic DNA was used directly for genotyping with 98 microsatellite (SSR) markers and 1 *Rc* gene‐specific marker (Subudhi et al., 2012) (Supporting Information Table S1). PCR amplification was performed with 0.1 µM labeled forward and reverse primers, 0.06 µM unlabeled dNTPs, 1× PCR buffer, and 0.08 units BioReady *Taq* polymerase (Bulldog Bio, Portsmouth, NH), and 10 ng DNA in a 8 µl PCR reaction. PCR reactions were run in a thermocycler with an initial denaturing step of 5 min at 94°C, followed by 35 cycles of 15 s at 94°C, 15 s at 55°C, and 30 s at 72°C, and a final extension of 5 min at 72°C. Products were resolved in a 6% polyacrylamide gel using an ABI 377 DNA sequencer (Applied Biosystems, Waltham, MA). Allelic differences between samples were scored based on allele size for genetic diversity (GenAlEx) and population STRUCTURE analyses. Genetic data were also scored as present (1) or absent (0) for each allele for the construction of phylogenetic trees.

To examine the biotypes of weedy rice existing in California and the southern United States, genetic diversity and differentiation indices, including the mean number of alleles detected per locus, Shannon diversity index, observed and expected heterozygosity, unbiased expected heterozygosity, and inbreeding coefficient were assessed for weedy rice biotypes (populations) using GenAlEx v6.5 software (Peakall & Smouse, 2006). Genetic differences among groups of California weedy rice were also inferred by conducting an analysis of molecular variance (AMOVA) in GenAlEx software.

To assess relationships between all rice samples, phylogenetic analysis of all 96 samples of California weedy rice, southern United States weedy rice, wild rice, and cultivated rice was conducted using neighbor‐joining analysis with 1,000 bootstrap iterations in DARwin v.6 software (Perrier, Flori, & Bonnot, 2003) with allelic data from 99 genetic markers. To assess the membership of individual genotypes into clusters allowing for genetic admixture, allelic genotype data were analyzed for genetic structure using STRUCTURE software (Pritchard, Stephens, & Donnelly, 20002000; Falush et al., 2003) using 98 microsatellite markers, excluding the *Rc* gene‐specific marker as STRUCTURE analysis is only appropriate for neutral genetic variation. Analysis was conducted with correlated allele frequencies and an admixture model for *K* values ranging from 1 to 20. Each run was conducted with a burn‐in period of 100,000 steps followed by 100,000 Monte Carlo Markov Chain replicates. To further assess the relationships of rice samples without assumptions of specific relationship or population models, a principal component analysis (PCA) of all samples was conducted in DARwin software using 99 genetic markers. In order to examine the level of differences or genetic relatedness among weedy rice biotypes and other rice groups as a whole, we looked at the pairwise fixation index (*F*_ST_) among clusters of wild rice, cultivated, and weedy rice biotypes.

## 3 RESULTS

The 99 markers used in this study covered the 12 chromosomes of rice with an average of 8 markers per chromosome and a mean interval distance of 4.43 Mb between markers. The markers showed high polymorphism with an average of 5 alleles and a mean polymorphism information content (PIC) value of 0.61 per marker. In total, 508 different alleles were scored among 96 rice genotypes using the 99 markers (Supporting Information Table S1). The presence or absence of a 14‐basepair deletion at the *Rc* gene correlated with red or white pericarp in rice individuals (Table 1). All weedy rice individuals had the wild‐type allele lacking the deletion, demonstrating the effectiveness of this marker for genetic identification of red pericarp in California weedy rice (Table 1).

**Figure 2:**
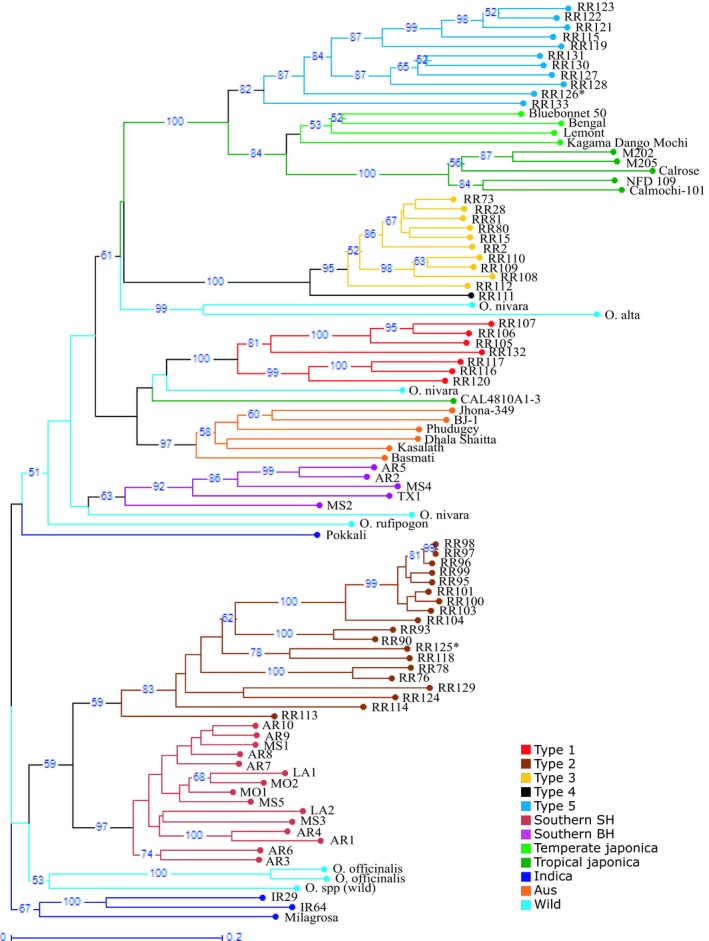
Phylogenetic tree showing relationships among 96 rice samples, including 46 California weedy samples, 20 weedy rice samples from the southern United States, 8 wild rice species, and 22 rice cultivars constructed using genotype data from 99 genetic markers. Bootstrap values higher than 50% are displayed

In the neighbor‐joining phylogenetic analysis, individuals largely clustered by rice type (Figure 2). While bootstrap support for many basal branches of the tree is low, the grouping of most rice individuals into clusters by rice type is well‐supported. The cultivated *japonica* rice varieties were separated from *indica* and all other rice groups with the exception of one red‐pericarped temperate *japonica* variety, indicating the effectiveness of SSR markers in differentiating the two main subspecies of rice. Moreover, the tropical and temperate *japonica* rice varieties are well separated. The *indica* rice samples, however, were not placed together, with IR29, IR64, and Milagrosa clustered together separately from red‐pericarped Pokkali landrace rice. Basmati rice, which is an aromatic rice belonging to group V rice, clustered with *aus* rice varieties with high bootstrap support. The wild rice species samples were scattered throughout the tree as expected by the wide diversity in their genomes. The wild rice *O. officinalis* samples, which have a CC genome type, clustered together. The southern United States weedy rice individuals were grouped into two separate clusters, with strawhull weedy rice clustered together with high bootstrap support, while blackhull weedy rice grouped together with less support.

The California weedy rice samples were grouped into four clusters, which correspond to five distinct biotypes categorized by hull color, grain type, and presence of awn (Figure 2, Table 2). The first cluster grouped all the short grain (SG), strawhull, awnless individuals (SH, called Type 1 hereafter). While high bootstrap values support the grouping of Type 1 individuals, the cluster is grouped near an *O. nivara* individual and one temperate *japonica* variety as well as *aus* and Basmati rice with low statistical support. The second California weedy rice cluster included all the medium grain (MG), bronzehull, and awnless weedy rice individuals (BrH, Type 2), placed near southern SH weedy rice and some wild rice. A third cluster grouped all the MG, strawhull with long awn (SHA+, Type 3). The single SG blackhull with long awn individual (BHA+, Type 4) closely grouped together with the strawhull Type 3 weedy rice. The fourth weedy rice cluster grouped together some MG and long grain (LG) strawhull weedy rice accessions with variable awn length (Type 5). Type 5 weedy rice was placed with high bootstrap support near the *japonica* rice varieties, and these two groups were placed with low support near Type 3 and Type 4 weedy rice. The clustering of California weedy rice by grain attributes validates the division of weedy rice samples by phenotypic similarities.

Two noncertified introduced cultivated red‐pericarped specialty rice varieties grown in California, (RR125 and RR126), clustered with California weedy rice. These red‐pericarped cultivated rice varieties have not gone through California's third‐party variety certification and inspection process and have been previously implicated in rice contamination (Timothy Blank, personal communication, 7 March 2017). RR125 clustered within Type 2 weedy rice and RR126 clustered within MG Type 5 weedy rice, indicating that these red‐pericarped specialty rice varieties are related to California weedy rices. It is unclear from this analysis, however, whether the California weedy rice could be derived directly from these noncertified varieties or whether their relationship is the result of gene flow from these varieties or their ancestors into another population. Since California weedy rice individuals clustered into distinct biotypes, genetic differences among groups of weedy rice were examined in more detail. Analysis of molecular variance (AMOVA) indicated that California weedy rice collections are very diverse, with the majority of the variation (55%) due to differences among groups (biotypes) while 40% is due to variation among individuals, and differences within group or biotype account for only 5% of genetic variation (Table 3). Each weedy rice biotype is genetically distinct from the others with an overall *F*_ST_ value of 0.548 among biotypes. Comparison of genetic diversity patterns among the four major biotypes (Table 4) indicate that Type 5 is the most diverse group with the highest number of alleles detected per locus (Na = 2.43), highest percentage of polymorphic loci (%PL), and most heterozygous alleles (He, uHe). In contrast, Type 3 weedy rice has the lowest number of alleles detected per locus (Na = 1.71), lowest Shannon diversity index within group (*I* = 0.29), and lowest number of heterozygotes. Type 2, which was found in four counties (Sutter, Yuba, Butte, Glenn), is also diverse (within‐group diversity, *I* = 0.52) but has the highest inbreeding coefficient (*F*_IS_) estimate of 0.90, indicating homozygosity of individuals in this group (Table 4). Overall, California weedy rice biotypes are genetically diverse but with a high frequency of homozygous alleles at 99 loci as indicated by high mean *F*_IS_ estimate for each group or biotype as well as the overall estimates of *F*_IS_ (0.888) and *F*_IT_ (0.949), as would be expected for a species such as rice that reproduces primarily by self‐fertilization.

**Table 3:** Summary of analysis of molecular variance and *F*‐statistics

Source of variation	*df*	SS	MS	Est. Var.	% Variance
Among biotype clusters (populations)	3	1,450.775	483.592	19.735	55
Among individuals	44	1,354.121	30.775	14.476	40
Within individuals	48	87.500	1.823	1.823	5
Total	95	2,892.396		36.035	100

*F*‐statistics	Value	*p*(rand perm. 999)
*F*_ST_	0.548	0.001
*F*_IS_	0.888	0.001
*F*_IT_	0.949	0.001

Note

*df*: degree of freedom; Est. Variance: estimated variance; *F*_ST_: overall genetic divergence among populations; *F*_IS_: overall inbreeding coefficient of individual within the population; *F*_IT_: overall inbreeding coefficient of an individual relative to the total population (individual within the total population); MS: mean square; *p*(rand perm. 999): significance of genetic distance after 999 random permutations; PhiPT: estimate of genetic distance among populations; SS: sum of squares; % Variance: percent variance.

**Table 4:** Mean genetic diversity indices among the four major biotypes of California weedy rice and two biotypes of southern United States weedy rice

	Type 1		Type 2		Type 3		Type 5		Southern SH		Southern BH	
	Mean	*SE*	Mean	*SE*	Mean	*SE*	Mean	*SE*	Mean	*SE*	Mean	*SE*
Na	1.94	0.10	2.40	0.12	1.71	0.09	2.43	0.12	1.64	0.09	1.768	0.08
I	0.47	0.04	0.52	0.04	0.29	0.03	0.61	0.04	0.27	0.03	0.384	0.04
Ho	0.04	0.02	0.02	0.01	0.03	0.01	0.06	0.02	0.02	0.01	0.04	0.01
He	0.30	0.03	0.30	0.02	0.17	0.02	0.36	0.03	0.16	0.02	0.241	0.02
uHe	0.32	0.03	0.31	0.03	0.18	0.02	0.38	0.03	0.17	0.02	0.269	0.03
*F*_IS_	0.84	0.04	0.90	0.03	0.83	0.04	0.83	0.04	0.86	0.04	0.806	0.04
% PL	63		77		55		75		43		57	
*N*	7		19		11		11		15		5	

Note

*F*_IS_: Inbreeding Coefficient = (He − Ho)/He = 1 − (Ho/He); He: Expected Heterozygosity = 1 − Sum pi^2^; Ho: Observed Heterozygosity = No. of Hets/*N*; *I*: Shannon diversity index = −1 × Sum (pi × Ln (pi)); uHe: Unbiased Expected Heterozygosity = (2*N*/(2*N* − 1)) × He; *N*: total number of individuals in a population (type); Na: no. of alleles detected per locus; % PL: percentage of polymorphic loci.

**Figure 3:**
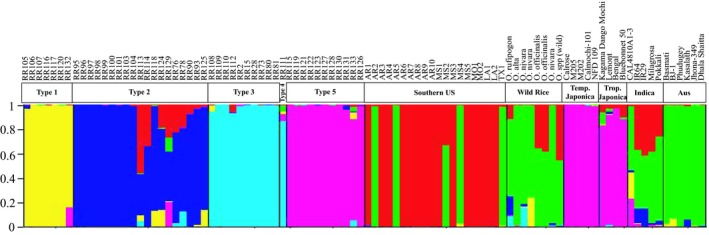
Individual genotype assignments of rice samples from population structure analysis of 98 microsatellite markers in STRUCTURE at *K* = 6 genetic clusters. The vertical bars represent the percentage of an individual's genotype assigning to each colored genetic cluster

To investigate the relationships among rice individuals while allowing for gene flow and admixture, unlike phylogenetic analysis, STRUCTURE analysis was used to assign each individual's genotype to genetic clusters or populations. The largest increase in data probability (Δ*K*) was observed at *K* = 6 (Supporting Information Figure S1) (Evanno, Regnaut, & Goudet, 2005), and this model distinguishes the major biotype groups fairly well (Figure 3). The STRUCTURE grouping of California weedy rice individuals and all other rice samples is consistent with their group membership from phylogenetic analysis (Figure 2). The majority of individuals assign to a single cluster with high probability, and most individuals of the same biotype assign to the same genetic cluster (Figure 3). However, the majority of *indica* rice and wild rice individuals assign to multiple clusters, indicating higher background genetic diversity or admixture between clusters. Some weedy rice individuals also assign to multiple clusters, indicating hybridization with or evolutionary origin from other rice groups. The cluster that all Type 1 individuals assign to also has minor genetic contributions from *O. nivara*, one *indica* rice variety, and some Type 2 weedy rice individuals. Some Type 2 individuals show admixture with strawhull weedy rice from the southern United States, *indica* rice, or wild rice species. Type 3 and Type 4 rice individuals all assign highly to a cluster that also has minor contributions from wild rice. Type 5 weedy rice individuals cluster genetically with both tropical and temperate *japonica* rice.

**Figure 4:**
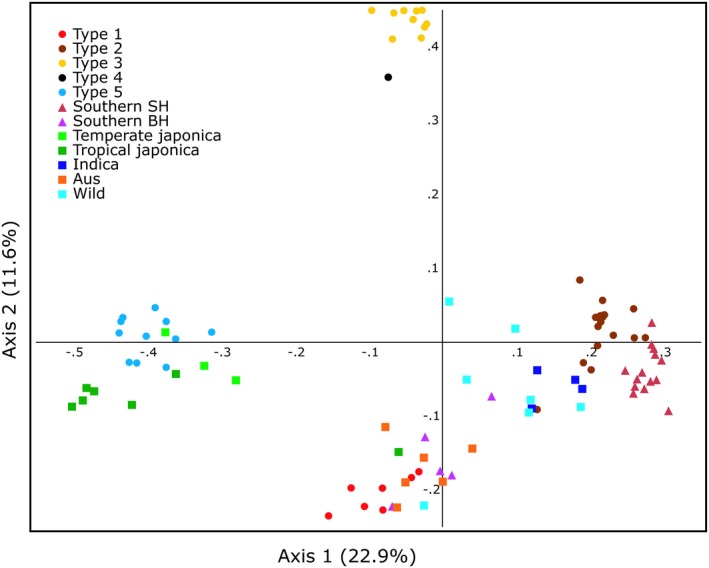
Genetic clustering of rice samples in principle component analysis using 99 genetic markers. Axes 1 and 2 account for 22.9% and 11.6% of the variation present

A principal component analysis (PCA) was used to assess genetic similarities among individuals without assuming specific relationship or population models (Figure 4). The first three axes account for 22.9%, 11.6%, and 10.2% of genetic variation present. As in previous analyses, most rice individuals cluster together by rice type, and were spatially well differentiated on the first two axes (Figure 4). Type 1 rice clustered closely with *aus* rice, Basmati, the single temperate *japonica* individual that clustered separately from the others in phylogenetic analysis (Cal4810A1‐3), and BH southern weedy rice. Type 2 weedy rice individuals clustered with SH southern weedy rice and *indica* rice. Type 3 and Type 4 rice clustered closely together, well differentiated on axis 2 from all other rice samples. Type 5 clustered together with temperate and tropical *japonica* rices. The wild rice samples did not cluster together closely, but were distributed mostly in the lower right corner. Genetic differentiation between biotypes was assessed for all weedy, wild, and cultivated rice biotypes. Most estimates of pairwise *F*_ST_ were high, ranging from 0.177 to 0.696 (Table 3). The very high pairwise *F*_ST_ values between the single Type 4 individual and all other groups indicate genetic differentiation but are likely artificially high due to the sample size of 1. The majority of California weedy rice biotypes, with the exception of Type 5, show high genetic differentiation (*F*_ST_ > 0.35) from the temperate *japonica* rice cultivars grown in California (Table 5). In contrast, low pairwise *F*_ST_ between a weedy rice biotype and another rice type can indicate more shared genetic content. For example, Type 2 shows low differentiation from *indica* cultivars (*F*_ST_ = 0.224) and from wild rice (*F*_ST_ = 0.214), indicating less differentiation between these groups and possible relatedness.

**Table 5:** Table of pairwise *F*_ST_ between groups of rice samples. Higher values indicate higher genetic differentiation between pairs of biotypes

	Type 1	Type 2	Type 3	Type 4	Type 5	Wild	S.BHA	S.SH	Temp.	Trop.	*indica*	*aus*
Type 1	0.000											
Type 2	0.373	0.000										
Type 3	0.544	0.439	0.000									
Type 4	0.619	0.537	0.473	0.000								
Type 5	0.396	0.383	0.472	0.567	0.000							
Wild	0.232	0.214	0.350	0.436	0.289	0.000						
S.BHA	0.335	0.371	0.544	0.627	0.391	0.238	0.000					
S.SH	0.505	0.293	0.587	0.696	0.476	0.257	0.454	0.000				
Temp.	0.367	0.395	0.484	0.561	0.247	0.285	0.407	0.507	0.000			
Trop.	0.476	0.472	0.567	0.659	0.339	0.345	0.488	0.556	0.264	0.000		
*indica*	0.359	0.224	0.449	0.570	0.371	0.177	0.362	0.281	0.368	0.425	0.000	
*aus*	0.265	0.342	0.494	0.610	0.377	0.223	0.327	0.454	0.357	0.458	0.322	0.000

Note

S. BHA: blackhull awned weedy rice from the southern United States; S.SH: strawhull weedy rice from the southern United States; Temp.: temperate *japonica*; Trop.: tropical *japonica*.

## 4 DISCUSSION

The increasing spread of weedy rice in California and the recent report of weedy rice originating from cultivated California rice varieties (Kanapeckas et al., 2016) raised questions about the origin of California weedy rice and its management. For this reason, we conducted a genetic study to understand the relationships between existing weedy rice in California and to investigate their possible origins. In the phylogenetic analysis, weedy rice individuals clustered together by biotype, indicating that for California weedy rice biotypes, samples can be easily classified by phenotype into groups that are biologically and genetically meaningful (Figure 2, Table 2). The five biotypes of California weedy rice clustered within multiple larger genetic groups of weedy, wild, and cultivated rice (Figure 2). This division of weedy rice into separate clusters most likely indicates at least four separate evolutionary origins of California weedy rice from diverse lineages of cultivated, weedy, and wild rice. In fact, the four major groups of weedy rice are quite divergent from each other based on principal component analysis (Figure 4). Population structure analysis gives more insight into relationships of individuals and biotypes, revealing close correspondence between genetic populations and rice types (Figure 3). However, some rice groups, especially wild rice and *indica* rice, are more genetically heterogeneous, with genotypes assigning to multiple genetic clusters. STRUCTURE analysis also identified admixed individuals, indicating hybridization of weedy rice both with other weedy rice biotypes and with wild and cultivated rice (Figure 3), despite the fact that rice is primarily self‐fertilizing with generally low outcrossing rates (0.4%–11%) (Xia et al., 2011).

Individual and biotype differentiation analyses provide insights into the relationships of California weedy rice biotypes. The high pairwise *F*_ST_ values between most California weedy rice biotypes, with the exception of Type 5, and the temperate *japonica* cultivars widely grown in California, indicates high genetic differentiation between California weedy rice and California cultivated rice and their relatively low shared genetic content (Table 5), suggesting that most weedy rice did not evolve from the cultivated rice varieties widely grown in California. The observed *F*_ST_ levels do not necessarily exclude the possibility of infrequent hybridization with cultivated rice within California. One Type 1 individual and one Type 2 individual showed over 10% genetic assignment to the genetic cluster containing Type 5 and *japonica* rices in STRUCTURE analysis (Figure 3). However, the majority of California weedy rice biotypes have a high inbreeding coefficient and low level of heterozygosity at 99 loci (Table 4). Therefore, it is likely that hybridization between rice groups happened many years or generations ago. Type 5 weedy rice was shown in phylogenetic, STRUCTURE, and PCA analyses to be closely related to *japonica* cultivars, raising questions of whether it is derived directly from the temperate *japonica* cultivars grown in California or from tropical *japonica* cultivars outside California and imported. The high inbreeding coefficient (*F*_IS_ = 0.83) of Type 5 weedy rice (Table 4) and moderate genetic differentiation (*F*_ST_ = 0.247) from temperate *japonica* rice (Table 5) make it likely that its evolutionary origin significantly predates its recent detection, although it is possible that a small weedy rice population could have been present unnoticed for some time prior to detection.

Another possibility for the origin and spread of California weedy rice is from the cultivation of red‐pericarped specialty rice varieties. While the majority of rice‐growing acreage in California is devoted to noncolored pericarp rice production, some specialty colored pericarp rice varieties are also grown at a commercial scale. Two noncertified specialty rice varieties (called RR125 and RR126 here) are medium grain rice with red pericarp. RR125 is similar to bronzehull Type 2 weedy rice while RR126 is a strawhull type that is mostly awnless. In phylogenetic analysis, RR125 was grouped with Type 2 weedy rice and RR126 was grouped with Type 5 weedy rice (Figure 2). STRUCTURE indicated membership of RR125 to the genetic cluster composed primarily of Type 2 weedy rice with a small amount of admixture from the cluster composed primarily of Type 1 weedy rice (Figure 3). RR126 assigned with 100% membership to the genetic cluster composed of Type 5 weedy rice and *japonica* rice varieties (Figure 3). Our evaluation of seed shattering and seed dormancy at 35–40 days after flowering indicated that RR125 has low shattering and no seed dormancy (100% seed germination), similar to cultivated rice (T. B. De Leon, unpublished data, 2017). RR126 is also nonshattering, but has high seed dormancy (2% seed germination) (T. B. De Leon, unpublished data, 2017). These results emphasize the need for a consensus definition of weedy rice, as not all red‐pericarped rices are weedy. However, the clustering of both RR125 and RR126 with weedy rice biotypes does indicate possible genetic contributions from red‐pericarped varieties into weedy populations. This potentially problematic relationship calls for careful management of rice seed and certification to avoid the contamination of white‐pericarped cultivated rice acreage with red‐pericarped specialty rice or its weedy relatives.

The genetic clustering of individuals by biotype observed in phylogenetic, STRUCTURE, and PCA analyses (Figures 1, 2, 3) indicate that phenotypic similarity is a better indicator of relatedness than geographic proximity for California weedy rice. The four major biotypes all contained samples from multiple different counties, and the only location where all individuals belong to the same biotype is Colusa County (Table 1). The spread of biotypes across a large geographic area is likely the result of seed movement, as pollen of both cultivated and wild rice travels only up to 100 m from source plants (Rong et al., 2010). The most likely mode of weedy rice seed movement over long distance is dispersal by humans, either through contaminated seed stocks or equipment within California or through additional introductions of weedy rice from other rice‐growing regions. This highlights the need for growing weed‐free certified seed in California and encouraging growers to prevent the spread of weedy rice on contaminated equipment. Recent regulations regarding the importation of used equipment and the requirement for only planting certified seed or seed from a third‐party quality assurance program should aid in these efforts (Prevention & Eradication of Weedy Rice, CA 3 CCR§, 2852.5, 2018).

Overall, the phylogenetic, population structure, and principal component analyses above allow some insights into the ancestry of California weedy rice and into the prevalence of evolutionary histories of de‐domestication. Type 1 weedy rice is likely evolutionarily derived from *aus* rice or possibly a wild rice species, as is the blackhull awned rice from the southern United States (Li et al., 2017). These two American weedy rice biotypes may have a single origin from Asian rice or separate origins followed by hybridization with each other. However, they are both genetically and phenotypically distinct, as Type 1 rice is neither blackhulled or awned (Table 2). Because the specific origins and any subsequent hybridization are unclear, it cannot be determined whether this biotype is derived from endoferal de‐domestication directly from the crop cultivars or exoferal de‐domestication through hybridization of cultivars and/or weedy populations. Type 2 weedy rice is most closely related to strawhull weedy rice from the southern United States, and these two groups likely evolved by exoferal de‐domestication from *indica* rice. Alternatively, these two groups were also placed with wild rice species in several analyses and could have some ancestry from undomesticated rice. Regardless, it is unclear whether Type 2 and southern strawhull weedy rice have a single or separate origins. Type 3 weedy rice of California is highly differentiated from other rice types and has ambiguous evolutionary origins. Based on its closest relationship in the phylogenetic analysis, it appears to have evolved from wild rice (Figure 2) and may have retained wild traits such as pubescence of leaves, presence of long awn, high seed dormancy, and shattering, consistent with the results of Londo and Schaal (2007) using a single California weedy rice genotype (RR28). In another study of Type 3 weedy rice by Kanapeckas et al. (2016), California strawhull weedy rice had the lowest mean population divergence (*ϕ*_st_) from *O. rufipogon* from South East Asia, but was interpreted as having diverged from California cultivated rice based on coalescent modeling analysis. More study of this weedy rice biotype may be needed to fully understand its evolutionary origins. Type 4 weedy rice is most likely descended from Type 3 weedy rice. Type 5 weedy rice is endoferally derived from *japonica* rice, and it is not clear whether the direct ancestor is tropical *japonica* rice or temperate *japonica* rice grown inside or outside of California. For all California weedy rice biotypes, the presence of the causative *Rc* allele for red pericarp associated with wild rice or landrace rice never selected for white pericarp means that genetic contributions of endoferal ancestry from landrace rice or exoferal ancestry from wild rice cannot be ruled out.

In conclusion, the five major California weedy rice biotypes are not all closely related to each other and have diverse parentage from several major lineages of cultivated rice and wild rice, as well as relationships with weedy rice from the southern United States. Most biotypes are likely derived from independent origins outside of California, although hybridization between biotypes or with local cultivars may contribute to the evolution of weedy rice populations. Future study of California weedy rice with sequence data may help elucidate the evolutionary relationships of weedy rice types with currently ambiguous origins. The recent rediscovery and rapid spread of multiple weedy rice biotypes with evolutionary origins outside of California highlights the need for management of current weedy populations and measures to prevent further introductions of weedy rice into California.

## CONFLICT OF INTEREST

None declared.

## AUTHOR CONTRIBUTIONS

T. B. De Leon conducted the laboratory and greenhouse research. E. Karn and T. B. De Leon performed data analysis and wrote the manuscript. W. Brim‐DeForest, K. Al‐Khatib, and T. B. De Leon designed the research. L. Espino, T. Blank, C. B. Andaya, and V. C. Andaya contributed to the materials and methods of the research study. All authors read and approved the final manuscript.

## Data Availability

Genotype data for this paper have been archived in the Dryad repository (https://doi.org/10.5061/dryad.9v60c13) and are accessible there. To protect rice grower's identities, weedy rice sampling locations will be provided to those who wish to sign a confidentiality agreement.
