# Supplementary material for: Genetic variation and possible origins of weedy rice found in California
Source: Ecol Evol. 2019 Apr 22;9(10):5835–48. doi: 10.1002/ece3.5167 (PMC6540678; doi:10.1002/ece3.5167)
Supplement: Supplementary file 2 [file ECE3-9-5835-s002.docx]

Supplemental Table S1. Distribution and allele variation of 98 microsatellite markers and 1 *Rc* gene-specific marker among 96 weedy, cultivated and wild rices. Microsatellite marker primer sequences are available on the Gramene database (http://archive.gramene.org/markers/microsat/). The *Rc* marker is published in Subudhi et al. 2012.

| SSR Marker | Chromosome | Physical Map Position (Mb)a | No. of Alleles | Polymorphism Information Content (PIC) |
| --- | --- | --- | --- | --- |
| RM84 | 1 | 1.00 | 7 | 0.78 |
| RM3426 | 1 | 4.05 | 4 | 0.66 |
| RM259 | 1 | 7.40 | 4 | 0.64 |
| RM579 | 1 | 8.45 | 6 | 0.77 |
| RM312 | 1 | 9.62 | 4 | 0.48 |
| RM10748 | 1 | 11.77 | 3 | 0.55 |
| RM2318 | 1 | 24.14 | 8 | 0.88 |
| RM306 | 1 | 24.50 | 5 | 0.74 |
| RM5781 | 1 | 35.76 | 7 | 0.73 |
| RM6405 | 1 | 29.53 | 3 | 0.32 |
| RM12146 | 1 | 40.71 | 7 | 0.77 |
| RM279 | 2 | 2.88 | 6 | 0.64 |
| RM5512 | 2 | 4.22 | 4 | 0.61 |
| RM6911 | 2 | 9.00 | 8 | 0.83 |
| RM5651 | 2 | 23.56 | 12 | 0.82 |
| RM341 | 2 | 14.95 | 6 | 0.79 |
| RM1367 | 2 | 27.05 | 5 | 0.68 |
| RM3512 | 2 | 27.31 | 9 | 0.71 |
| RM5631 | 2 | 26.43 | 7 | 0.66 |
| RM208 | 2 | 35.13 | 5 | 0.69 |
| RM166 | 2 | 29.79 | 2 | 0.17 |
| RM266 | 2 | 33.05 | 5 | 0.70 |
| RM3203 | 3 | 0.79 | 8 | 0.65 |
| RM3894 | 3 | 1.11 | 2 | 0.32 |
| RM3392 | 3 | 3.82 | 5 | 0.70 |
| RM5819 | 3 | 4.28 | 4 | 0.45 |
| RM5513 | 3 | 6.28 | 5 | 0.60 |
| RM7 | 3 | 10.52 | 4 | 0.64 |
| RM487 | 3 | 22.01 | 3 | 0.73 |
| RM6329 | 3 | 28.80 | 8 | 0.78 |
| RM3564 | 3 | 33.42 | 4 | 0.10 |
| RM335 | 4 | 0.68 | 4 | 0.53 |
| RM8213 | 4 | 4.44 | 9 | 0.77 |
| RM3742 | 4 | 19.74 | 2 | 0.25 |
| RM1359 | 4 | 19.86 | 9 | 0.67 |
| RM3866 | 4 | 23.17 | 5 | 0.79 |
| RM3785 | 4 | 24.06 | 8 | 0.78 |
| RM3836 | 4 | 31.62 | 4 | 0.65 |
| RM5506 | 4 | 33.30 | 5 | 0.69 |
| RM131 | 4 | 34.42 | 3 | 0.62 |
| RM3531 | 4 | 35.21 | 7 | 0.81 |
| RM1366 | 5 | 2.91 | 5 | 0.69 |
| RM164 | 5 | 19.19 | 5 | 0.68 |
| RM7568 | 5 | 19.42 | 3 | 0.46 |
| RM31 | 5 | 28.61 | 4 | 0.67 |
| RM1054 | 5 | 29.16 | 3 | 0.39 |
| RM469 | 6 | 0.56 | 4 | 0.64 |
| RM225 | 6 | 3.41 | 5 | 0.77 |
| RM3431 | 6 | 8.74 | 3 | 0.68 |
| RM527 | 6 | 9.86 | 4 | 0.71 |
| RM3827 | 6 | 22.29 | 4 | 0.71 |
| RM6298 | 6 | 23.73 | 4 | 0.50 |
| RM340 | 6 | 28.59 | 9 | 0.82 |
| RM439 | 6 | 29.62 | 6 | 0.62 |
| RM345 | 6 | 30.86 | 4 | 0.69 |
| RM5463 | 6 | 30.98 | 4 | 0.86 |
| RM6652 | 7 | 0.58 | 2 | 0.38 |
| RM436 | 7 | 2.55 | 2 | 0.49 |
| RM5711 | 7 | 3.10 | 7 | 0.72 |
| Rc1_T | 7 | 6.00 | 2 | 0.18 |
| RM214 | 7 | 12.78 | 2 | 0.76 |
| RM505 | 7 | 19.50 | 3 | 0.29 |
| RM3826 | 7 | 20.80 | 6 | 0.68 |
| RM234 | 7 | 25.47 | 7 | 0.71 |
| RM346 | 7 | 13.84 | 3 | 0.47 |
| RM5720 | 7 | 28.66 | 11 | 0.88 |
| RM248 | 7 | 29.33 | 4 | 0.67 |
| RM408 | 8 | 0.12 | 3 | 0.82 |
| RM3689 | 8 | 19.33 | 11 | 0.78 |
| RM44 | 8 | 13.44 | 7 | 0.73 |
| RM149 | 8 | 24.72 | 7 | 0.78 |
| RM3496 | 8 | 27.83 | 6 | 0.79 |
| RM5799 | 9 | 3.80 | 5 | 0.69 |
| RM24245 | 9 | 13.76 | 2 | 0.33 |
| RM3700 | 9 | 15.42 | 7 | 0.74 |
| RM205 | 9 | 22.72 | 6 | 0.76 |
| RM244 | 10 | 0.87 | 3 | 0.60 |
| RM216 | 10 | 5.35 | 5 | 0.76 |
| RM1146 | 10 | 19.61 | 5 | 0.68 |
| RM590 | 10 | 23.04 | 3 | 0.69 |
| RM286 | 11 | 0.38 | 6 | 0.68 |
| RM26167 | 11 | 3.86 | 7 | 0.83 |
| RM4504 | 11 | 5.47 | 2 | 0.85 |
| RM202 | 11 | 9.00 | 7 | 0.78 |
| RM3701 | 11 | 8.10 | 1 | 0.00 |
| RM287 | 11 | 16.76 | 4 | 0.70 |
| RM206 | 11 | 22.01 | 10 | 0.81 |
| RM144 | 11 | 28.28 | 4 | 0.74 |
| RM224 | 11 | 30.00 | 7 | 0.82 |
| RM1208 | 12 | 0.00 | 3 | 0.59 |
| RM1808 | 12 | 0.74 | 6 | 0.51 |
| RM5927 | 12 | 2.20 | 8 | 0.87 |
| RM3455 | 12 | 4.19 | 6 | 0.94 |
| RM7619 | 12 | 4.82 | 4 | 0.57 |
| RM3103 | 12 | 7.46 | 3 | 0.70 |
| RM313 | 12 | 9.64 | 3 | 0.33 |
| RM270 | 12 | 22.75 | 8 | 0.71 |
| RM6947 | 12 | 23.97 | 2 | 0.40 |
| RM1226 | 12 | 27.31 | 5 | 0.58 |
| Average: | 8.2^b^ | 4.43^c^ | 5.13^d^ | 0.61^e^ |
| ^a^marker position based on gramene.org | | |  |  |
| ^b^mean number of markers/chromosome | | |  |  |
| ^c^mean distance between two adjacent markers | | |  |  |
| ^d^mean number of alleles per marker | | |  |  |
| ^e^mean PIC per marker. | |  |  |  |
